# Maternal brain reactive antibodies profile in autism spectrum disorder: an update

**DOI:** 10.1038/s41398-023-02335-3

**Published:** 2023-02-03

**Authors:** Ciara Bagnall-Moreau, Benjamin Spielman, Lior Brimberg

**Affiliations:** 1 Institute of Molecular Medicine, The Feinstein Institutes for Medical Research, Northwell Health System, Manhasset, New York, NY USA; 2 Donald and Barbara Zucker School of Medicine at Hofstra/Northwell, Hempstead, NY USA

**Keywords:** Autism spectrum disorders, Molecular neuroscience

## Abstract

Autism spectrum disorder (ASD) is a heterogeneous neurodevelopmental disorder with multifactorial etiologies involving both genetic and environmental factors. In the past two decades it has become clear that in utero exposure to toxins, inflammation, microbiome, and antibodies (Abs), may play a role in the etiology of ASD. Maternal brain-reactive Abs, present in 10–20% of mothers of a child with ASD, pose a potential risk to the developing brain because they can gain access to the brain during gestation, altering brain development during a critical period. Different maternal anti-brain Abs have been associated with ASD and have been suggested to bind extracellular or intracellular neuronal antigens. Clinical data from various cohorts support the increase in prevalence of such maternal brain-reactive Abs in mothers of a child with ASD compared to mothers of a typically developing child. Animal models of both non-human primates and rodents have provided compelling evidence supporting a pathogenic role of these Abs. In this review we summarize the data from clinical and animal models addressing the role of pathogenic maternal Abs in ASD. We propose that maternal brain-reactive Abs are an overlooked and promising field of research, representing a modifiable risk factor that may account for up to 20% of cases of ASD. More studies are needed to better characterize the Abs that contribute to the risk of having a child with ASD, to understand whether we can we predict such cases of ASD, and to better pinpoint the antigenic specificity of these Abs and their mechanisms of pathogenicity.

## Introduction

Autism spectrum disorder (ASD) is a heterogeneous neurodevelopmental disorder with a very strong male bias. According to the Centers for Disease Control and Prevention, ASD affects ~1 in every 44 children in the United States. The core symptoms of ASD include social interaction impairments, communication deficits, repetitive behaviors, and restricted interests [1].

The etiology of ASD has been studied extensively in the past three decades. While it is clear that ASD has a genetic component, the contribution of the environment and especially the in utero environment has become an area of increasing interest. De novo variants may account for 2% of ASD cases [2, 3]. Heritability in ASD is in the range of 60–90% [4, 5]. Studies have estimated that common variants explain up to 50% of heritability [3]. The same studies estimate environmental and maternal contribution to be up to 35% [4, 5]. Environmental exposures associated with ASD tend to occur during the prenatal period, such as air pollution exposure, maternal infection, microbiome and autoimmune diseases (For review see [6, 7]). Some of the assumptions that are made in studies modeling the relative contribution of genetics and environment are, of necessity, oversimplified, and are not borne out in animal models. For example, maternal environment is sometimes considered the same even when considering monozygotic twins and maternal full or half siblings [4].

Given the considerable phenotypic and genetic heterogeneity, and the rising incidence, a multifactorial etiology involving both genetic and environmental factors may account for the pathology in ASD [8].

Murine models targeting ASD risk genes, such as the CNTNAP2 deficient mouse [9], recapitulate core behavioral deficits of ASD. Similar behavioral deficits are also observed in mouse models of ASD induced by environmental risk factors, including exposure to maternal inflammation [10–13], toxins [14], and maternal anti-brain antibodies (Abs) [15–19].

Support for the importance of the in utero environment in ASD pathogenesis comes from the observation that siblings born 18 months or less after the birth of a child with ASD have a higher risk for ASD than do siblings born four or more years later [20]. The assumption is that the closer in time siblings are born the more similar the environment is. Thus, increased sibling recurrence risk with an interbirth interval of 18 months or less, compared to an interval of 4 years or more suggest that the in utero environment has an effect even beyond genetic risk. Furthermore, maternal half-siblings of a child with ASD are at higher risk for ASD than are paternal half-siblings [20]. Given that paternal half siblings are likely to have the same genetic similarity as maternal half siblings but are less likely to be concordant for ASD suggest that the maternal (in utero) environment contributes to risk.

Moreover, women with autoimmune diseases have an increased risk for a child with ASD [21–24]. Mothers with diseases such as rheumatoid arthritis, celiac disease, and systemic lupus erythematosus (SLE) are more likely to have a child with ASD [22, 23, 25]. Since these diseases are mediated by auto-Abs, it has been hypothesized that maternal Abs can pose a risk for ASD.

### Maternal antibodies during fetal development

During development, maternal Abs have a crucial role in providing the fetus with passive protection against pathogens. Maternal Abs, specifically the Immunoglobulin G (IgG) subclasses IgG1 and IgG4, cross the placenta beginning around the second trimester of pregnancy [26, 27]. In brief, maternal IgG internalized by pinocytosis binds neonatal fragment crystallizable (Fc)-receptors (FcRn) within the acidic environment of endosomes of placental syncytiotrophoblasts [28, 29]. The FcRn–IgG complexes are then shuttled away from the lysosomal pathway, and IgG is released into the fetal circulation upon fusion with the plasma membrane [30–32]. The efficiency of maternal IgG transfer depends on maternal levels of total IgG, IgG subclass, gestational age, and placental integrity.

Since the blood brain barrier (BBB) is not fully formed in the developing fetus, maternal Abs present in fetal circulation can penetrate the brain parenchyma. Specifically, in mice, maternal IgG can enter the brain between embryonic days E12.5 and ~E16.5. By E17.5 the fetal BBB excludes IgG [33]. Although no study to date has shown the presence of maternal antibody in human embryonic brain, and this would be challenging to show due to difficulties in obtaining tissue for study, it is assumed that an analogous time course is present in humans and that maternal IgG can gain access to the developing human fetal brain during the second trimester [34]. Therefore, if the mother has Abs that target fetal brain antigens, these Abs might affect brain development, while the mother will not be affected since she has a mature BBB. The severity of the insult by maternal IgG may be determined by several factors such as the antigenic specificity, the localization and gestational stage at which the antigen is expressed, the level of the maternal IgG, as well as the sex of the fetus. For example, in a maternal SLE model, exposure in utero to a subset of lupus anti-DNA/N-methyl-D-aspartate (NMDA) receptor maternal IgG resulted in long-term cognitive impairments in male offspring [35], but death of female fetuses [36]. It was determined that the sex differential responses to the maternal IgG stemmed from the earlier temporal expression and abundance of the target antigen in the female brainstem [36]. The maternal IgG subclass is also an important mediator of the insult’s severity, as the downstream effector mechanism triggered by IgG depends on its subclass [37]. The pathogenic effect of in utero exposure to anti-aquaporin-4 (Aqp4) IgG1 cloned from a patient with Neuromyelitis Optica depends on the presence of the non-glycosylated form of Aqp4 in the embryonic brain and is mediated by complement-dependent cytotoxicity, since pathology is absent in mice lacking the complement component C1q [38].

In utero exposure to pathogenic maternal Abs has been shown to affect brain development in rodent models in cases in which mothers have underlying autoimmune conditions such as SLE [36, 39] or Neuromyelitis Optica [38]. However, pathogenic maternal Abs may also be present in a subgroup of healthy mothers, which raises the question as to what triggers their production and how they avoid the negative selection of autoreactive B cells.

One of the main functions of B cells is to produce Ab molecules to neutralize and destroy invading pathogens and their toxins. To this end, a diverse repertoire of Abs is required. The Ab repertoire is generated by random recombination of gene segments that results in diverse antigen-recognition sites during the immature phase of a B cell in the bone marrow. Since this process may result in B cells producing autoreactive Ab (immunoglobulin), there are immune check points in place that remove autoreactivity from the B cell repertoire, such that if the Ab on the cell surface of the B cells is binding to self-antigen, it will be eliminated by processes known as clonal deletion and receptor editing prior to becoming a mature B cell (for review see [40]). Additional autoreactive Abs can be generated once B cells encounter an antigen and differentiate further (also known as affinity maturation) in a process of somatic hypermutation which leads to point mutations in the binding region of immunoglobulin. While this can create a more effective protective Ab response, it can also generate auto-Ab. B cells producing autoreactive Ab that emerge during the hypermutation process are generally eliminated through lack of positive selection mechanisms. It is possible given that many central nervous system (CNS) specific antigens are sequestered, and their presentation is restricted in the periphery due to the BBB, CNS-reactive B cells escape negative selection at the time when the repertoire of B cells is established. CNS-reactive B cells may then be triggered to produce antibodies when encountering CNS antigens due to insults such as stroke or due to structural similarity between bacterial or viral antigens and brain antigens, a phenomenon known as molecular mimicry [41, 42].

In the past decade, different anti-brain Abs have been associated with ASD. They have been mainly suggested to bind neuronal antigens and may bind extracellular [15, 43] or intracellular antigens [44]. In this review we summarize the data from clinical and animal models studies supporting the role of pathogenic maternal Abs in ASD.

## Clinical data: maternal brain-reactive Abs and ASD

Clinical observations demonstrating an association between familial autoimmunity and ASD date back to 1971, when Money et al. discussed a possible relationship between a family’s history of autoimmune disease and the youngest child’s ASD diagnosis [45]. From the observations in this early study, the author hypothesized that the child’s ASD could be the “primary effect of autoimmune impairment from the formation of autoantibodies affecting the central nervous system.” Now, half a century later, there is considerable clinical data connecting maternal auto-Ab production to the development of ASD in their offspring.

Several early studies used western blotting techniques to characterize maternal Ab reactivity against brain proteins. One study demonstrated that postnatal serum taken years after pregnancy from mothers of a child with ASD (*n* = 11) can be distinguished from serum of mothers of a typically developed child (*n* = 10) by its binding patterns to fetal rat brain proteins on a western blot [46]. However, this study is limited by a small sample size and the use of postnatal rather than mid-gestational maternal serum. Croen et al. [47] studied 84 mothers of a child with ASD and 160 control mothers of a typically developed child and observed that serum collected from mothers of a child with ASD between gestational weeks 15–19 has different binding patterns to fetal brain proteins on western blot [47]. Reactivity to proteins of 39 kDa was more likely to be found in serum from mothers of a child with ASD than from mothers of a typically developed child (7.1% vs 2%, respectively), and reactivity to proteins of both 39 kDa and 73 kDa was exclusively observed in 3 of the 84 mothers of a child with ASD. In another study, postnatal serum was obtained from 100 mothers of a child with ASD and 100 mothers of an unaffected child, and western blot was used to test for reactivity to fetal and adult antigens derived from the brain tissue of humans and rats [48]. The researchers were able to identify differences in serum reactivity to human and rat fetal and adult whole brain homogenate. They also noted differences in serum binding to homogenates obtained from specific regions of the adult human brain, namely the cingulate gyrus, cerebellum, caudate, and to a defined area in the frontal cortex, Brodmann area 9. Despite using postanal serum and not identifying the specific antigenic proteins in each region of the brain, this study provided insight that there may be unique targets of maternal Abs in different regions of the brain that may contribute to ASD.

Further studies identified and characterized two protein bands as highly specific for mothers of a child with ASD [49]. In a study of maternal plasma collected from 61 mothers of a child with ASD, 40 mothers of a child with non-ASD developmental delays, and 62 mothers of a typically developed child, 12% of mothers of a child with ASD showed reactivity to 37 kDa and 73 kDa protein from human fetal brain protein on western blot, while no binding to these protein bands was observed in any of the other mothers. In a study using a larger cohort, reactivity to both protein bands at these molecular weights was observed in 7% of mothers of a child with ASD (*n* = 143), compared to 0% of mothers of a child with non-ASD developmental delays (*n* = 62) and mothers of a typically develop child (*n* = 121). The latter study further demonstrated that among 275 mothers of a child with the diagnosis of full autism or children with a broader ASD phenotype, the presence of maternal IgG reactivity to fetal brain proteins of 37 and 73 kDa correlated with lower expressive language scores, and the reactivity to 39 and 73 kDa correlated with higher irritability scores, compared to the children with ASD with mothers with no detected anti-brain reactivity [50].

We have previously used immunohistochemistry methodologies to detect maternal anti-brain Abs [51]. We screened postnatal plasma from mothers of a child with ASD (*n* = 2431) and from an unselected group of women (*n* = 653) and assessed the presence of anti-brain IgG using brain sections from adult male mice. Plasma from 10.5% of mothers of a child with ASD demonstrated strong binding to the adult brain sections, compared to plasma from 2.6% of control mothers. Thus, our study used a larger cohort to strengthen the findings from previous studies and provided further justification to study the role of maternal anti-brain Abs in the development of ASD. Moreover, although in this study we had used plasma taken years after pregnancy, in a follow-up prospective study we found a strong association between the presence of mid-gestational and postnatal maternal anti-brain Abs (unpublished data).

As the association between maternal anti-brain Abs and ASD becomes more established, many groups have begun to elucidate possible targets of these Abs. Studies from Braunschweig et al. were among the first to identify specific proteins bound by maternal anti-brain Abs from mothers of an ASD child [44]. They used 2D gel electrophoresis and mass spectrometry to identify specific maternal Ab reactivity to lactate dehydrogenase A and B (LDHA, LDHB), guanine deaminase (GDA), stress-induced phosphoprotein 1 (STIP1), collapsin response mediator proteins 1 and 2 (CRMP1, CRMP2), and Y-box-binding protein 1 (YBX1). Subsequent research added neuron-specific enolase (NSE) to the group of proteins recognized by the maternal Abs and characterized the specific antigenic peptide epitopes within these proteins that correlate with an increased risk of having a child with ASD [52, 53]. Having Abs targeting only one of these proteins is not specific for mothers of a child with ASD; 89% of mothers of a child with ASD (*n* = 246) have plasma reactivity to these proteins, compared to 70% of mothers of a typically developed child (*n* = 149) [44]. However, maternal Ab reactivity to specific combinations of these proteins (CRMP1 + GDA, CRMP1 + CRMP2, and NSE + STIP1) was more likely to be present in mothers of a child with ASD. 23% of mothers of a child with ASD had reactivity to one or more combination of those antigens, as opposed to only 1% of mothers of a typically developed child.

A follow up study verified the significance of having Abs against two or more of these proteins [54]. In this study, plasma reactivity to combinations of the previously mentioned eight proteins (LDHA, LDHB, GDA, STIP1, CRMP1, CRMP2, YBX1, and NSE) were assessed by an enzyme-linked immunosorbent assay (ELISA) in 540 mothers of a child with ASD, 184 mothers of a child with intellectual disability without ASD, and 420 control mothers from the general population. It was determined that 10% of mothers of a child with ASD displayed reactivity to two or more of these proteins, compared to 4% of mothers of a child with intellectual disability and 1% of the general population. When comparing mothers of a child with ASD to control mothers, reactivity to several combinations of proteins (CRMP1 + CRMP2, CRMP2 + STIP1, LDHA + YBOX, GDA + YBOX, CRMP1 + STIP1) was significantly more common in mothers of a child with ASD [54].

The discrepancies in the abundance and pattern of the maternal Ab reactivity between Braunscweig et al. [44] and Ramirez-Celis et al. [54] could stem from the use of postnatal [44] versus midgestational [54] serum, but more likely from the different techniques that were used. The higher prevalence in Braunscweig et al. [44] may be attributable to the less stringent conditions of the western blot and the high concentration of the protein that was used. Nevertheless, this combinatorial approach to measuring maternal Abs reactivity is a promising way to increase the predictiveness of these eight proteins as a biomarker. Indeed, the frequency of these maternal Abs is similar across cohorts from three different states (California, Pennsylvania and Arkansas) and may be related to worse ASD outcome [55].

Although the eight proteins (LDHA, LDHB, GDA, STIP1, CRMP1, CRMP2, YBX1, and NSE) have established roles in neurodevelopment, including in synaptogenesis, neuronal differentiation, neuronal survival, and other processes [56–69], each of the eight proteins is not specific to the brain, which raises a question of how these maternal auto-Abs are not harmful to other developing tissues or to the mother. For instance, LDHA and LDHB are predominantly found in muscle tissue and are known for their role in metabolism [56]. Furthermore, it is not clear how maternal Abs targeting these intracellular proteins can gain access to their targets in the absence of inflammation. One speculation is that circulating Abs against intracellular antigens can be up taken by cells through a receptor-mediated mechanism [70, 71]. It is also possible that during naturally occurring apoptosis in the fetus, immune complexes are formed with neuronal apoptotic material exposing intracellular proteins. This potentially could explain how these Abs are not harmful to the mother. It would be valuable to confirm the presence of those Abs in the brain parenchyma in animal models, and to confirm that insults to the fetal brain are not due to peripheral effects of these maternal Abs.

Other protein targets have been identified in maternal Ab-driven ASD. Our group isolated and characterized monoclonal brain-reactive Abs from the blood of women with anti-brain IgG and a child with ASD [15] and identified contactin-associated protein-like 2 (Caspr2) as one of the antigens targeted by the monoclonal Abs isolated in that approach. Caspr2 is a neuronal transmembrane protein that plays a role in neuronal migration, axonal growth and glutamate receptor membrane localization during development [9, 72, 73]. Mutations in CNTNAP2, the gene encoding Caspr2, have been associated with neurological disorders such as ASD, intellectual disability, schizophrenia, and dyslexia [74]. We demonstrated that 37% of mothers who harbor anti-brain Abs and have a child with ASD have Abs against Caspr2, as opposed to 12% of unselected women of childbearing age and 7.6% of mothers of a typically developed child [15]. Reactivity to Caspr2 in this study was assessed using a cell-based assay, in which maternal serum was analyzed for its binding to HEK-293T cells expressing Caspr2. This assay tests reactivity to the protein in its naive form.

A subsequent study reported that maternal anti-Caspr2 Abs are associated with intellectual disability in children, but not with ASD [75]. However, this study was not designed to assess whether mothers of children with ASD that harbor anti-brain Abs are more likely to harbor anti-Caspr2 Abs. Given their small cohort (95 mothers), it would be expected that 4 mothers harbor anti-brain Abs and anti-Caspr2 Abs. Their finding then does neither refute nor confirm the previous study [15]. Notably, this group reported that exposure in utero to these Abs leads to social deficits in a rodent model (see below) [43]. Further clinical analysis is necessary to assess the association between maternal anti-Caspr2 Abs and children with ASD.

Several auto-Abs more commonly associated with paraneoplastic syndromes have also been associated with maternal Ab-mediated ASD. In a study using western blot to assess postnatal maternal serum, mothers of a child with ASD were significantly more likely to harbor anti-Yo Abs (with an odds ratio of 2.6) and anti-amphiphysin Abs (odds ratio: 2.5) than were healthy mothers with a typically developed child [76]. A later study, using ELISA, confirmed the association between maternal anti-amphiphysin Abs and ASD, and found a significant association between anti-Ri Abs and ASD [77]. The differences in the findings of these studies could stem from the differences in sensitivity between immunoblotting and ELISA techniques. These differences could also be the result of the small sample sizes used in each study. Ali et al. [76] analyzed serum from 49 mothers of a child with ASD and 73 mothers of a typically developed child, while Bilgen Ulgar and colleagues [77] sampled 33 mothers of a child with ASD and 27 mothers of a typically developed child. It is of note that the study by Ali et al. [76] did not exclude mothers with autoimmune disease, whereas the study by Bilgen Ulgar et al. did. Performing a similar analysis on a larger cohort of mothers with more consistent exclusion criteria would give better clarity on the true relationship of these auto-Abs to ASD.

The relevance of anti-Ri and anti-Yo Abs in ASD remains to be determined. It is interesting to note that these antibodies are associated with paraneoplastic cerebellar degeneration, often secondary to various types of cancer including carcinoma of the breast or fallopian tube, adenocarcinoma in an axillary lymph node, and others [37, 78, 79]. The cerebellum has been implicated in social behavior, and changes in cerebellar function have been observed in both rodent models and humans with ASD [80–82]. Uptake of anti-Ri and anti-Yo antibodies has been observed in Purkinje cells in cerebellar slice cultures [83, 84], which might explain how these Abs targeting intracellular antigens can trigger a pathologic process leading to ASD, although the mechanism of their internalization is not understood.

Maternal Abs targeting non-neuronal antigens may also be involved in the pathogenesis of ASD. A study of mid-gestational maternal serum from a large Finnish cohort (960 mothers of a child with ASD and 960 mothers of a child without ASD) demonstrated that mothers of a child with ASD are 1.78 times more likely to harbor antibodies against thyroid peroxidase (TPO) than are mothers of a child without ASD [85]. In a subsequent study, it was observed that male offspring born to mothers harboring anti-TPO Abs are about twice as likely to develop ASD [86]. The mechanism by which anti-TPO Abs may lead to ASD is unclear. Female mice immunized against TPO have been shown to display a reduced preference for sucrose and longer immobility time on the tail suspension and forced swimming tests, all of which are used to assess depressive-like behaviors in rodents [87–89]. It has also been shown that females harboring anti-TPO Abs have reduced brain-derived neurotrophic factor and serotonin (5-HT) production in the prefrontal cortex, providing further evidence that anti-TPO antibodies may have effects on the CNS [87]. However, there is as yet no mechanism described for the link between maternal anti-TPO Abs and the risk of having a child with ASD.

## Animal models of maternal Abs induced ASD

Epidemiologic studies in humans have suggested a possible association between maternal auto-Abs and increased risk for ASD in the offspring. Since the presence of auto-Abs may not necessarily mediate pathogenesis, to demonstrate causality, investigators have utilized two approaches. The first is demonstrating that transfer of the auto-Abs from a mother of an ASD child into animals will produce pathologic features of ASD. This model usually includes isolation of the auto-Abs from the serum of the mother and injecting them into dams at mid gestation. The second involves immunization of female mice prior to pregnancy with self-antigen. In this model, female mice are immunized with the corresponding antigen that the human auto-Abs target to elicit the endogenous production of auto-Abs. These auto-Abs are then present throughout gestation. Both models, passive transfer and immunization, have been utilized in the studies described below. These approaches are needed to demonstrate the potential pathogenicity of auto-Abs in general, not just the potential pathogenicity of brain-reactive Abs. The ultimate confirmation of their pathogenicity is to demonstrate that their removal from maternal serum reduces risk of ASD, but this is not yet feasible.

### Non-human primates

Evidence for the pathological role of maternal Abs has been investigated in non-human primate models. In an early passive transfer study, pregnant female rhesus macaques received intravenous injections during the first trimester of their pregnancies of IgG that had been purified from mothers of a child with ASD. Rhesus macaques exposed in utero to purified IgG from mothers of a child with ASD exhibited a higher frequency of stereotypic behaviors and hyperactivity as measured by an increase in pacing movement episodes, compared to offspring exposed to control IgG from mothers of a typically developing child [90]. There were no profound differences in social behaviors of macaque offspring including maternal-infant interactions or post-weaning juvenile contact between the treatment groups. In this study, the pregnant female rhesus macaques received intravenous injections limited to a narrow window during the first trimester, which may not reflect the typical duration throughout pregnancy, nor the optimal time which IgG cross the placenta. Therefore, a major caveat of this early investigation is the timing of exposure and potentially the reduced effect of human IgG binding to macaque antigen, potentially explaining the modest effects of in utero Ab exposure on the social behaviors in the offspring [90].

Maternal anti-brain Abs reactive to 37/73 kDa antigens (IgG-ASD^37/73kDa^) associated with an ASD diagnosis in the child [44] were evaluated in a passive transfer model in rhesus monkeys. Prenatal exposure to these patient-derived Abs resulted in atypical primate social behavior as measured by more frequent approaches to familiar versus unfamiliar peers. Further neuroimaging analysis of male offspring revealed significant differences in brain growth, as offspring exposed in utero to the IgG-ASD^37/73kDa^ display larger total brain volume, particularly in the white matter of several brain regions at 2 years of age [91]. These findings are consistent with observations of larger brain volume in male children with ASD born to mothers with IgG-ASD^37/73kDa^ [92]. The mechanism by which maternal IgG-ASD^37/73kDa^ affects neurodevelopment remains poorly understood.

There are unique translational advantages to the use of non-human primates in preclinical ASD research owing to their close phylogenetic relationship to humans including similar physiology, neuroanatomy, and behavioral complexity. There are, however, several limitations in considering non-human primates as bona fide models for studying maternal Abs in ASD. In the aforementioned studies, the findings from individual behavioral or neuroimaging assessments were based on a relatively small sample size of rhesus macaques, owing to the limited availability of offspring [90, 91]. Furthermore whereas altered behaviors were found in both male and female offspring exposed in utero to IgG-ASD^37/73kDa^, MRI neuroimaging analyses revealed significant developmental differences in brain growth only in males, [91]. The latter finding is, however, in accordance with human studies suggesting that cerebral enlargement is mostly limited to boys with ASD [92].

### Rodent studies

An early study by Dalton et al. [18] explored a maternal-fetal passive transfer model, administering serum from a mother of a child with ASD or pooled sera from mothers of a typically developed child into pregnant mice starting at gestational age E10. The authors found lower exploratory activity and reduced performance on the multiple static rod test suggesting impaired motor coordination in offspring exposed in utero to serum of a mother of a child with ASD. Furthermore, a reduction in the levels of the cerebellar metabolites creatine and choline was observed using magnetic resonance spectroscopy in the offspring of dams injected with serum from a mother of a child with ASD compared to offspring of dams injected with serum from mothers of a typically developed child, implicating the role of the cerebellum in the pathophysiology of ASD. Although the antigenic targets associated with the behavioral changes in the offspring were not identified in this study, the serum was demonstrated to bind Purkinje cells in the cerebellum and a surface antigen expressed on unpermeabilized neuroblastoma cells. Singer et al. [93] further demonstrated that maternal Abs can lead to neurodevelopmental alterations in mice. In contrast to earlier reports using whole serum, this study evaluated the behavioral consequences of offspring born to dams that received daily injections of purified IgG pooled from 63 mothers of a child with ASD or IgG from mothers of a typically developed child, starting from gestational age E13. Adolescent offspring exposed in utero to Abs obtained from mothers of a child with ASD were found to exhibit sociability deficits and anxiety-like behavior as measured by performance on the elevated plus maze, changes that persisted into adulthood [93].

The pathological relevance of maternal IgG-ASD^37/73kDa^ was also evaluated in a gestational passive transfer rodent model. Similar to the study in rhesus monkeys, Braunschwieg et al. [94] administrated maternal IgG binding the 37/73 kDa proteins from mothers of a child with ASD to pregnant mice. Juvenile offspring born to these dams, compared to offspring born to dams administered IgG from mothers of a typically developed child, demonstrated delayed sensory and motor development, as well as a higher frequency of ultrasonic vocalizations in male pups, suggestive of increased anxiety [94]. In a series of studies, maternal IgG-ASD^37/73kDa^ was administered once, intraventricularly, into mouse embryos at gestational day E14 to evaluate the direct effects of exposure to IgG-ASD^37/73kDa^ on brain development. Using this alternative approach of IgG delivery into the developing brain, maternal IgG-ASD^37/73kDa^ was found to bind radial glial cells in the cortex and to lead to increase in neuronal precursor cell number. Exposure in utero to IgG-ASD^37/73kDa^ also led to an increase in brain size in male adult offspring [95]. Despite the brain pathology seen in male mice exposed in utero to IgG-ASD^37/73kDa^, IgG-ASD^37/73kDa^ exposed female, but not male mice, were observed to have atypical social interactions as measured by interaction with a novel object and a stranger mouse in the three-chamber social interaction test. Females, but not males, were also observed to have an increase in repetitive behavior, as measured by increased grooming duration [96]. Alterations to the dendritic arborization and dendritic spine density of basal dendrites of neurons in the cortical infragranular layers were found in IgG-ASD^37/73kDa^ adult offspring when combined both sexes together compared to control IgG; it is not clear if these treatment group differences were specific to changes in either male or female offspring, or both [97].

The finding that IgG-ASD^37/73kDa^ binds LDH-A, LDH-B, STIP1, and CRMP1 [44, 49, 52, 98] led to further studies addressing the impact of these maternal Abs under more physiological settings. In contrast to the passive transfer methodology, female mice were immunized prior to pregnancy against peptide sequences corresponding to epitopes of LDH-A, LDH-B, STIP1, and CRMP1, which partially reflects the originally observed IgG-ASD^37/73kDa^ banding pattern [17]. These four peptides were selected because human clinical data showed that maternal reactivity to these four proteins was the most common combination of auto-Abs exclusively found in mothers of a child with ASD [91]. In the maternal immunization approach, dams harbor endogenously produced pathogenic Abs throughout gestation, similar to the human condition. Juvenile and adult offspring born to dams immunized with the 37/73 kDa peptide mixture were subject to extensive behavioral phenotypic tests to evaluate developmental milestones and to assess anxiety, social interaction, and stereotypic behaviors. Female, but not male, pups born to dams immunized with the 37/73 kDa peptide mixture emitted overall more ultrasonic vocalizations (USVs) than control offspring in response to social isolation. Overall, male and female juvenile mice had fewer social interactions with a same-sex juvenile mouse and increased repetitive self-grooming bouts. Increased grooming behavior was evident also in adult male and female offspring born to dams immunized with the 37/73 kDa peptide mixture. Adult males also displayed deficits in reciprocal social interactions (reduced sniffing and time following) with an unfamiliar female mouse in estrus, and a reduced number of USVs were emitted during the social interactions. The adult female offspring were not assessed in a reciprocal social interaction paradigm and had no social deficits in the three-chambered sociability task (i.e, showed normal preference for interacting with a novel mouse over an object). Despite the comprehensive screening of the offspring behavioral profile, Jones et al. did not include neuropathological assessment.

In a subsequent neuroanatomical study using ex-vivo Magnetic Resonance Imaging (MRI) on fixed brains, Bruce et al. [99] reported an increase in the total and regional brain volume of female, but not male offspring born to dams immunized with the 37/73 kDa peptide mixture. When assessing how the regional brain volumetric changes are related to one another, the authors found that only male, but not female, offspring born to dams immunized with the 37/73 kDa peptide mixture displayed reduced connectivity within the cortex and hippocampus. Further analysis revealed a correlation between discrete brain regions and associated juvenile reciprocal social interaction behaviors in females, specifically between the number of nose-to- anogenital sniffing behavior and the regional volume of the bed nucleus of stria terminalis and nucleus accumbens. Male offspring showed a correlation between the dorsal lateral orbital cortex and repetitive self-grooming behavior [99].

Although the authors describe a correlation between the juvenile behavior deficits and adult regional brain size, contrasting observations were made in male and female offspring. The molecular mechanisms by which exposure to maternal IgG-ASD^37/73kDa^ could result in sex- differential effects on brain structure and behavior remain unclear. Nonetheless, the exploration of antigen-driven immunization models should offer mechanistic insights into the effects of maternal Abs on neurodevelopment and ASD risk.

### Maternal anti-Caspr2 Abs

Only one extracellular antigen implicated in maternal Ab-associated ASD has been identified. We have cloned antibodies from mothers with a child with ASD and found that these Abs can bind the extracellular portion of both human and mouse Caspr2. We have first used a passive transfer of the Ab, administering dams at embryonic day E13.5 with the human monoclonal Caspr2 IgG (termed C6) [15]. The male fetuses exposed in utero to C6 IgG exhibit early brain abnormalities including a thinner cortical plate and fewer proliferating cells in the ventricular zone at E15.5. In adulthood, male mice exposed in utero to C6 IgG also exhibit focal cortical changes, including fewer neurons in the entorhinal cortex. These adult offspring exhibit a decreased complexity of dendrites on excitatory neurons, and reduced numbers of the inhibitory parvalbumin-expressing interneurons in the CA1 region of the hippocampus. Furthermore, these mice demonstrate ASD-like behaviors including deficits in sociability, impaired flexible learning, and stereotypic behaviors. In a follow-up study, we have developed a new model in which females were immunized with the extracellular portion of human Caspr2 and generated high titers of polyclonal anti-human and anti-mouse Caspr2 Abs [16]. Results of studies of offspring exposed in utero to these Abs, were similar to the effects observed in mice exposed in utero to C6; male offspring exposed in utero to polyclonal anti-Caspr2 IgG exhibit brain abnormalities and ASD-like behaviors in adulthood. This is the only model in which the maternal Abs and the antigen have been unequivocally identified and is the only model with a strong sex bias.

Additional groups have investigated the pathogenic effects of anti-Caspr2 IgG exposure in a maternal–fetal transfer model, administering purified anti-Caspr2 IgG derived from sera of a male patient with Caspr2 autoimmune encephalitis to dams, daily, starting from embryonic day E12.5 [43]. In their clinical study described earlier, Coutinho et al. suggested that maternal anti-Caspr2 Abs do not associate with ASD in children but rather are more frequent in mothers of a child with mental retardation [75]. Surprisingly, they showed that in utero exposure to these patient-derived anti-Caspr2 Abs leads to long-term adverse consequences in affected male and female mouse offspring including social interaction deficits and abnormal lamination of neurons in the somatosensory cortex at adulthood [43]. Furthermore, these mice also have a reduction in the expression of glutamatergic synaptic proteins and a concomitant increase in the activation of microglia, suggesting that in utero exposure to anti-Caspr2 Abs can lead to pruning of synapses and contribute to the behavioral impairments associated with ASD [91, 93].

Observations of offspring exposed in utero to monoclonal human or polyclonal mouse anti-Caspr2 IgG reveal a sex-specific impact on behavior and brain development that aligns with the strong male preponderance documented in ASD [15, 16]. Although the precise mechanisms by which maternal anti-Caspr2 IgG leads to neurodevelopmental phenotypes in the offspring are not yet fully evaluated, we have speculated that anti-Caspr2 IgG binding to Caspr2 activates microglia through Fc receptor engagement. A previous study has shown that exposure in utero to IgG purified from plasma of patient with high titers to Caspr2 resulted in increased activation of microglia [43]. It is possible that that microglia activation starts early in development and persists into adulthood and that microglia suppression may ameliorate the ASD-like phenotype.

## Mechanism of maternal Abs induced ASD

One of the main caveats of studies supporting the role of maternal brain-reactive Abs in ASD is a lack of mechanism. Below we describe two possible mechanisms by which Abs may mediate its effect in the developing brain.

**Fig. 1:**
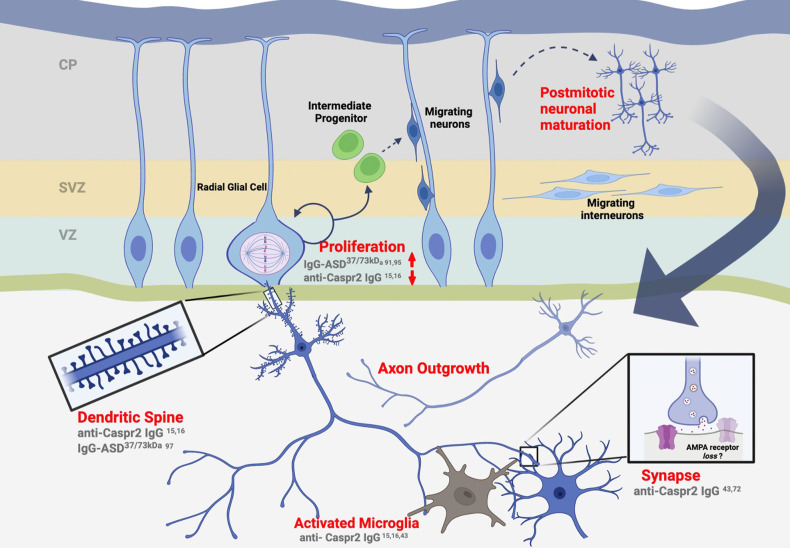
Summary of the abnormalities in the embryonic and postnatal mouse brain altered by brain-reactive Abs of mothers of a child with ASD. Abs are listed in gray. Figure was created with Biorender.com.

First, the antigen binding region of the Ab (Fab) can potentially mediate pathology by altering the function of the targeted molecules. Abs to extracellular molecules can block normal cellular interactions or can activate downstream signaling cascades, modulating neuronal function. This mechanism would trigger a specific pathology depending on the molecule the Ab targets (Fig. 1).

Second, Abs can cause inflammation through the effector functions of the Fc region of the Ab molecule, by complement dependent cytotoxicity (CDC) or Ab-dependent cell-mediated cytotoxicity (ADCC). To date, no study has reported on cell death mediated by maternal brain-reactive Ab in ASD, as would occur with either CDC or ADCC. Alternatively, complement may be utilized to recruit microglia and enable phagocytic activity and pruning. The Fc region of antibody can also engage Fc receptors, activating Fc receptors on microglia, triggering the release of inflammatory mediators. In the developing fetal brain, all components of the complement cascade can be engaged, and Fc receptors are present on microglia [65, 100]. Microglial activation and pruning alterations have been reported in models of maternal brain-reactive Ab induce ASD [15, 16, 43, 96].

Fc receptor dependent mechanisms could be a common mechanism for maternal Abs induced ASD, as they are independent of the target antigen, potentially leading to a common therapeutic approach to maternal brain-reactive Ab induced ASD.

## Discussion

**Fig. 2:**
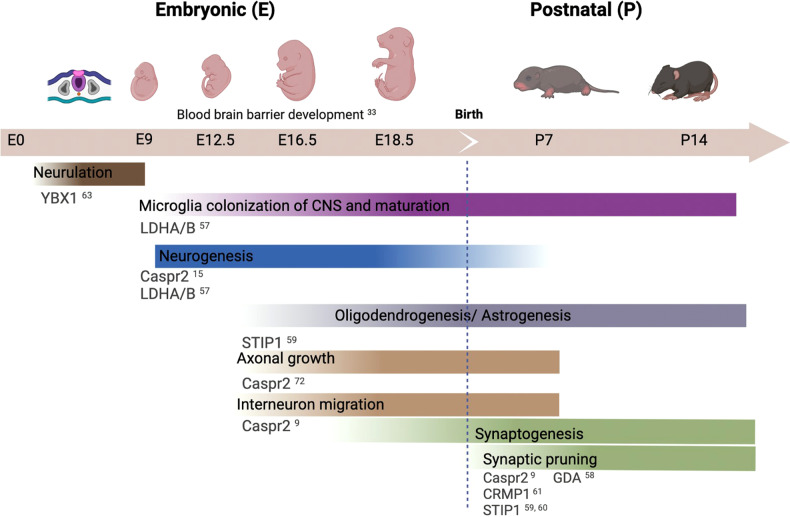
Neurodevelopmental timeline of antigens targeted by Abs of mothers of a child with ASD. Schematic of processes occurring during prominent periods of embryonic and postnatal brain development in mice. The antigens hypothesized to be involved in the neurodevelopmental processes are listed in dark gray. Figure was created with Biorender.com.

Maternal Abs represent a potential environmental risk factor for ASD. Exposure in utero to maternal brain-reactive Abs may alter the fetal brain and result in neurodevelopmental disorders (Fig. 2). Several groups have shown that the presence of maternal anti-brain Abs is correlated with increased risk of having a child with ASD and have demonstrated the harmful potential of these Abs to the developing brain in animal models. Several antigenic targets of these maternal Abs have been identified, although the specific pathogenic mechanisms related to ASD remain unclear.

Animal studies testing the effects of endogenous production of Abs following immunization, or the effects of direct administration of serum or purified IgG isolated from a mother of a child with ASD into non-human primates or dams, have provided compelling evidence supporting a pathogenic role of brain-reactive Abs. Overall, these studies have demonstrated that the placental transfer of maternal Abs into the fetus alters the fetal brain tissue, and have confirmed a causal role for maternal Abs in neuroanatomical and behavioral changes associated with ASD [15, 17, 93, 94].

Not all Abs targeting brain antigens are likely to affect brain development; mothers harboring anti-brain Abs can still give birth to a typically developed child. This discrepancy may reflect Ab titers, or a requirement for a particular Ab specificity or affinity. It may also stem from differences in posttranslational modifications of the antigen such as glycosylation of the protein during development and specificity of maternal Abs for the glycosylated protein, as has been shown for anti-Aqp4 Ab [38]. Alternatively, it may suggest that additional factors including maternal microbiota, maternal inflammatory responses, or fetal genetics might modulate the effects of anti-brain Abs on risk for developmental disorders in children. The evidence is, however, strong that a subset of ASD is the result of maternal Abs affecting biological processes during critical periods of brain development.

It is important to note that sex differences in this etiology of ASD have not been sufficiently investigated, and clinical studies have focused primarily on findings in male children. In the animal models of maternal Ab-associated ASD, there are inconsistencies in the behavior and neuroanatomical outcomes of male and female offspring, likely due to variations in antibody specificity, timing of exposure, delivery method, and species differences. For example, in the non-human primate studies, although both male and female offspring display behavioral deficits, only male offspring exposed in utero to IgG^ASD37/73kDa^ form a mother of a child with ASD were found to exhibit differences in brain growth and white matter volume as assessed by neuroimaging analyses [91]. However, in a later study using ex vivo MRI, increased brain volume was seen only in female, but not male offspring born to dams immunized with the corresponding 37/73 kDa peptide mixture.

The different mouse models of IgG-ASD^37/73kDa^ also do not consistently replicate the core features of ASD in male and female offspring. For example, although alterations in social interactions and stereotypic behaviors were observed among offspring exposed to IgG-ASD^37/73kDa^ compared to control offspring, these differences are attributable to effects on female and not male offspring [96]. We observed a significant male bias in neuroanatomy and behavioral measures in offspring exposed in utero to anti-Caspr2 IgG [15, 16], although no sex differences were found in placental transfer of these antibodies into male and female fetuses or in the expression level of the target antigen [16].

Maternal anti-brain reactive Abs are a particularly overlooked promising field of research as they may account for up to 20% of cases of ASD [44, 51, 53], representing a modifiable cause of ASD. For example, blocking maternal antibodies from binding antigens in the fetal brain can be achieved by fusing the target antigen or its critical epitope to an Fc region that does not cross the placenta. We have previously shown that specific mutations to the Fc region of IgG1 can both prevent transplacental transfer, and at the same time reduce downstream effector mechanisms such as CDC [38]. Thus, administrating a fusion protein consisting of antigen and mutated Fc during pregnancy to absorb pathogenic Ab could theoretically remove pathogenic maternal Abs without affecting the developing fetus. Alternatively, Bolandparvaz et al. [101] developed Systems for Nanoparticle-based Autoantibody Reception and Entrapment, which potentially could bind specific circulating maternal Abs, without triggering innate or complement immune response. In this method, target antigen or epitope-coated Dextran Iron Oxide Nanoparticles are administrated to the mother during pregnancy; Abs recognizing the antigen bind the nanoparticle and are cleared with, presumably, no subsequent effect on the fetus.

Despite over a decade of research many questions remain to be answered: How do anti-brain Abs emerge in healthy mothers? What is the spectrum of anti-brain Abs that increase the risk of ASD in the offspring? What are their mechanisms of action? Can they be used as early biomarkers to detect ASD? Are males more susceptible to the harmful effects of the maternal anti-brain Abs?

More studies need to be done to better characterize the mothers that are at risk of having a child with ASD, to understand whether we can we predict such cases of ASD and to better pinpoint the antigenic specificity of these Abs and their mechanism of pathogenicity.
